# Association and predictor role of MASC scores in pharmacological or psychological treatment indication in a sample of children and adolescent in Spain

**DOI:** 10.1192/j.eurpsy.2024.957

**Published:** 2024-08-27

**Authors:** C. Canga-Espina, C. Vidal-Androher, M. Vallejo-Valdivielso, C. Maestro-Martin, A. Diez-Suarez

**Affiliations:** ^1^Child and Adolescent Psychiatry; ^2^Clinical Psychology, Clínica Universidad de Navarra, Pamplona, Spain

## Abstract

**Introduction:**

Anxiety is one of the most common Mental Health diagnosis in underage population. We decided to study if there was any variable that would lead us to a specific treatment indication using the MASC (Multidimensional Anxiety Scale for Children).

**Objectives:**

Prevalence of psychiatric disorders and comorbidities in an underage population.

Possible association between MASC questionnaire scores and the indication for pharmacological and/or psychological treatment.

**Methods:**

This is a descriptive, observational, retrospective, quantitative study with data from patients between June 2016 and 2023. Inclusion criteria: 3-18 year-old-spanish-speakers who met criteria for a ICD-11 disorder. Exclusion criteria: absence of legal representatives, intellectual disability. **Variables:** Age, sex, psychiatric family history, ICD-11 diagnosis, treatment indication and MASC’s subscales (physical symptoms, harm avoidance, social anxiety and separation anxiety). **Statistical analyzes** were performed with STATA-15 program, using as independent variables MASC questionnaire, and dependent ones the indication treatment and diagnosis.

**Results:**

The sample contains 1024 patients, with a mean age of 12 (SD 4.028). Table 1 shows that the most frequent diagnosis is ADHD, with combined presentation with a prevalence of 22.27%, followed by Anxiety Disorders, without differentiating by subtypes (17.93%). It also shows that Defiant and Oppositional Disorder is the most prevalent comorbidity (9.66%) followed by Anxiety Disorder not specified (4.99%). Table 2 stands that there are significantly higher scores in all MASC subscales in those patients who do have prior psychiatry family history. We founf in Table 3 statistically significant differences were found between the score on the Physical Symptoms subscale based on whether the patient was undergoing previous treatment, both pharmacological (8.45 vs. 7.59) and psychological treatment (9.01 vs. 7.95) compared to those who were not (pharmacological 7.36 vs. 7.06), psychological (7.21 vs. 6.92). All these data have been adjusted.

**Image:**

**Figure:**
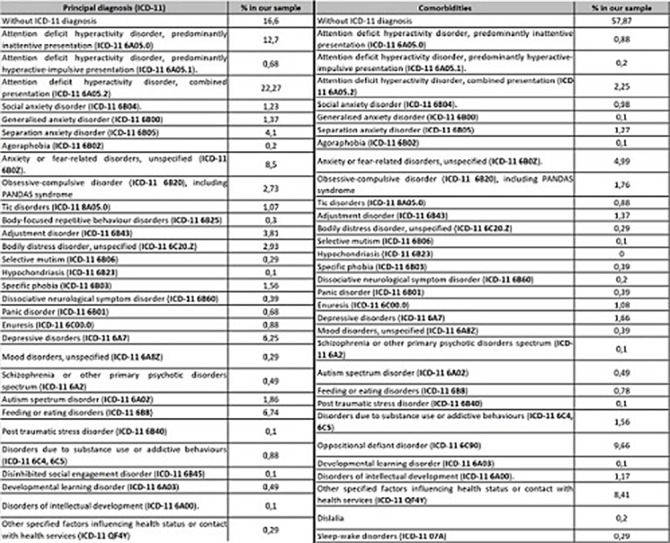

**Image 2:**

**Figure:**
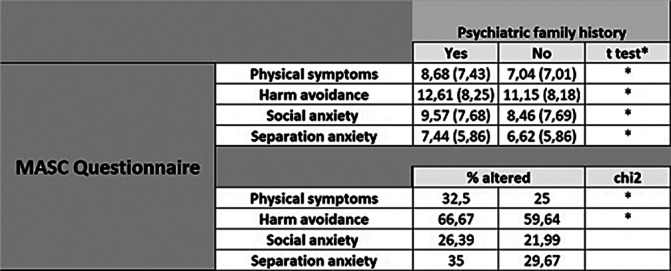

**Image 3:**

**Figure:**
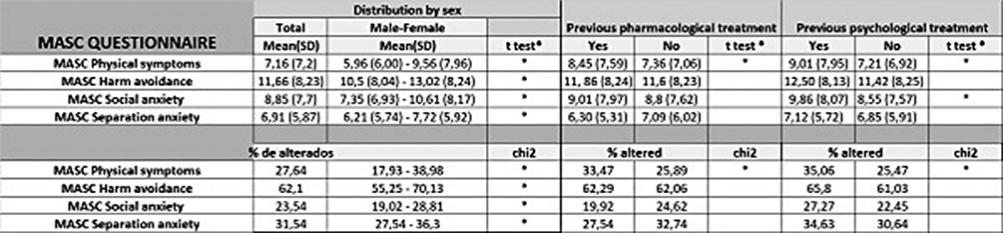

**Conclusions:**

Anxiety disorders are the most common form of Mental Disorder in young people, with a global prevalence of 6.5% (Rapee et al.2023). However, in our sample the most common one is ADHD as our center is specialized in it. We found that the most prevalent one was Oppositional Defiant Disorder, as it is the most frequent comorbidity of ADHD (Vallejo-Valdivielso et al,2019; Faraone et al,2021). The increase of one point in the Physical Anxiety subscale increases the probability of indicating pharmacological treatment, which could be explained because of how functional limitation these symptoms cause. The increase in all the subscales of the MASC implies an increase in the probability of an indication for psychological treatment as it is the gold-standard treatment for anxiety in children.

**Disclosure of Interest:**

None Declared

